# The crystal structure of the toxin EspC from enteropathogenic *Escherichia coli* reveals the mechanism that governs host cell entry and cytotoxicity

**DOI:** 10.1080/19490976.2025.2483777

**Published:** 2025-03-31

**Authors:** Akila U. Pilapitiya, Lilian Hor, Jing Pan, Lakshmi C. Wijeyewickrema, Robert N. Pike, Denisse L. Leyton, Jason J Paxman, Begoña Heras

**Affiliations:** a Department of Biochemistry and Chemistry, La Trobe Institute for Molecular Science, School of Agriculture, Biomedicine and Environment, La Trobe University, Bundoora, Australia; b Research School of Biology, Australian National University, Canberra, Australia

**Keywords:** Enteropathogenic *E. coli*, autotransporter protein, serine protease, toxins, bacterial infections, secretion system, diarrhoea

## Abstract

Enteropathogenic *E. coli* (EPEC) is a significant cause of diarrhea, leading to high infant mortality rates. A key toxin produced by EPEC is the EspC autotransporter, which is regulated alongside genes from the locus of enterocyte effacement (LEE), which collectively result in the characteristic attaching and effacing lesions on the intestinal epithelium. In this study, we present the crystal structure of the EspC passenger domain (α^EspC^) revealing a toxin comprised a serine protease attached to a large β-helix with additional subdomains. Using various modified EspC expression constructs, alongside type III secretion system-mediated cell internalization assays, we dissect how the α^EspC^ structural features enable toxin entry into the intestinal epithelium to cause cell cytotoxicity.

## Introduction

Diarrheal diseases, including dysentery, pose significant health problems, especially in developing countries, where they cause over 1.3 million deaths annually among children under five and contribute to substantial healthcare costs.^1–3^ Pathogenic *Escherichia coli* and *Shigella* are two major enteric pathogens responsible for these diseases. A critical factor that increases the pathogenicity of these diarrheal pathogens is the secretion of high molecular weight toxins known as serine protease autotransporters of *Enterobacteriaceae* (SPATEs). SPATEs are categorized into two classes: class 1, which damage the intestinal epithelium and class 2, which facilitate bacterial colonization through mechanisms such as mucin degradation and modulation of the immune response.^4–6^

SPATEs are part of the largest family of secreted and outer membrane proteins from Gram-negative bacteria, known as autotransporters.^7,8^ Autotransporters form the type V secretion pathway and share a common architecture that allows them to facilitate their own secretion. This includes an N-terminal signal peptide for transport into the periplasm and a C-terminal translocator domain that forms a pore in the outer membrane, allowing the secretion of a central functional passenger domain to the bacterial surface.^9–11^ Notably, the BAM complex is required for insertion of the autotransporter translocator domain into the outer membrane.^12^

SPATE passenger domains, as revealed by experimental structures, exhibit high structural conservation, with an N-terminal trypsin-like serine protease domain (subdomain-1/SD1), followed by an extended right-handed 3-stranded β-helix.^5,9^ Loop extensions from the β-helix can form two subdomains (SD2–3). After transport to the bacterial surface, the SPATE passengers are released into the extracellular environment.

Many of the class 1 SPATEs enter human tissue to cause cell rounding and, ultimately, disruption to the intestinal epithelium and inflammation, contributing to symptoms of enteric infections such as diarrhea. Plasmid encoded toxin (Pet) from enteroaggregative *E. coli* (EAEC) is the most comprehensively studied of these class 1 SPATEs.^13^ The Pet subdomain-2 (SD2) interacts with the epithelial receptor cytokeratin-8 to enter cells by receptor-mediated endocytosis, where it enters the cell cytosol via trafficking in clathrin-coated vesicles.^14,15^ Upon entry into the cytosol, the Pet protease subdomain cleaves actin-associated fodrin to cause cellular rounding and disruption to the epithelium.^16^

The SPATE class 1 enterotoxin EspC is associated with enteropathogenic *E. coli* (EPEC), a major cause of severe and sometimes fatal diarrhea in infants, particularly in developing countries.^17^ Since EPEC lacks the ability to produce Shiga toxins, heat-labile and heat-stable enterotoxins,^17^ EspC is considered a significant toxin in this pathogen. EspC is encoded in the second pathogenicity island (PAI) of EPEC,^18^ but its expression is regulated by the Ler regulator together with genes in the enterocyte effacement (LEE) pathogenicity island. The latter is responsible for intimate colonization, enterocyte effacement, and pedestal formation leading to the characteristic lesions of EPEC infection.^19^ The LEE pathogenicity island exerts these effects through the injection of effectors into host cells via a type III protein secretion system (T3SS) encoded on the LEE.^20,21^

The secreted passenger domain of EspC (α^EspC^) plays a central role in the cytopathic effects on intestinal epithelium during EPEC infection. Similar to Pet, α^EspC^ enters epithelial cells where its serine protease subdomain (SD1) cleaves cell cytoskeleton-associated fodrin as well as cell attachment proteins paxillin and focal adhesion kinase, to cause cell rounding and detachment.^22,23^ However, EspC appears to have several defining attributes that set it apart from other autotransporters. The α^EspC^ can enter epithelium on its own, but its interaction with the type III secretion system accelerates and enhances its entry. As such, EspC remains the only autotransporter to utilize two major protein secretion systems, type III and V for bacterial secretion and cellular translocation.^24,25^ In addition to causing cell rounding, α^EspC^ can also induce cell death by activating the apoptosis and necrosis pathways.^26^

In this study, we have elucidated the mechanisms by which the structural features of the EspC passenger domain (α^EspC^) contribute to its role in EPEC infections. Through determining the crystal structure of α^EspC^ and a detailed structure-function analysis, we have systematically dissected how specific structural elements of α^EspC^ enable its efficient entry into intestinal epithelial cells, leading to cytotoxic effects. This comprehensive approach offers new insights into the molecular interactions underlying EspC activity, which could pave the way for potential therapeutic interventions against EPEC-mediated diarrheal diseases.

## Materials and methods

### Bacterial strains, plasmids, and growth conditions

Bacterial strains and plasmid constructs used in this study are listed in Table S1. All strains were routinely grown aerobically in Luria-Bertani (LB) broth media or LB agar supplemented with 100 µg mL^−1^ ampicillin.

### Molecular cloning and construction of EspC mutants

Mutants of EspC were generated by mutagenesis using the Q5 Site-Directed Mutagenesis Kit (New England Biolabs, USA) with pBAD30:*espC* as the template.^23^ The oligonucleotides used are listed in Table S2, and all constructs were confirmed by dideoxy nucleotide sequencing (Macrogen Inc., Korea).

### Expression and purification of α^EspC^

Plasmids encoding wildtype or mutant EspC were transformed into *E. coli* Top10 cells (Invitrogen), and cells grown aerobically in LB broth with 100 μg mL^−1^ ampicillin to OD_600_ of ~1. Protein expression was induced by adding 0.02% (w/v) L-arabinose for 24 h at 30°C for wildtype EspC or 20°C for EspC mutants. The culture supernatants containing secreted proteins (secreted passenger domains) were obtained by centrifugation at 8,000 × *g* for 30 min, filter sterilized (VacuCap 90 PF filter device; Pall Life Sciences, Australia), and concentrated 100-fold (Vivaflow 200, 30 kDa MWCO, Sartorius, Germany). The concentrated supernatants were dialyzed into 100 mM sodium acetate, pH 4–5, before cation exchange chromatography using HiTrap SP Fast Flow column (GE Healthcare) with 50 mM sodium acetate (pH 4–5) and proteins eluted with a linear gradient to 500 mM NaCl. The pH of cation exchange buffers ranges from pH 4–5, depending on the isoelectric point of each α^EspC^ variant. Each protein was further purified by gel filtration chromatography using a Superdex 200 Increase 10/300 GL column (GE Healthcare) pre-equilibrated with phosphate-buffered saline (PBS). The purity of the fractions was analyzed by SDS-PAGE gels and Western blotting with α^EspC^ polyclonal antisera (1:25000). Protein concentrations were determined using a Nanodrop 2000 spectrophotometer (Thermo Fisher Scientific, USA), and proteins were flash-frozen in liquid nitrogen and stored at −80°C.

### Crystallization and diffraction data measurement

Purified α^EspC^ (13 mg mL^−1^ in 25 mM HEPES, pH 7.0, 150 mM NaCl) was crystallized in 0.1 M Bis-Tris propane pH 7.4, 0.2 M potassium citrate and 15% (w/v) PEG 3350 using the hanging-drop vapor-diffusion technique. Harvested crystals (after 27 d incubation at 20°C) pre-equilibrated in reservoir solution containing 25% glycerol were flashed-cooled in liquid nitrogen.

Diffraction data for α^EspC^ were collected on the micro-crystallography beamline MX2 at the Australian Synchrotron. The reflection data were collected using an Eiger detector (Dectris, Baden-Dättwil, Switzerland) at a wavelength of 0.9537 Å with a crystal-to-detector distance of 360 mm and a total angular rotation of 360° with an oscillation range of 0.1° per frame.

### Structure determination of native α^EspC^

Diffraction data were indexed, integrated and scaled with HKL2000 software,^27^ and the α^EspC^ was solved by molecular replacement using Phaser^28^ within the CCP4 suite^29^ and the structure of EspP passenger domain (PDB:3SZE)^30^ as a model. A model of α^EspC^ was completed by manually building Coot^31^ and refinement using Refmac5^32^ and Phenix refine^33^ within the CCP4 suite. The structure quality was validated by the MolProbity^34^ server and all the structural figures were generated by the molecular graphics tool PyMOL.^35^ Details of data-processing statistics and final refinement values are summarized in Table 1.

**Table 1.** αEspC data collection and refinement statistics

**Parameter**	**Native αEspC**
**Data collection**	
Detector	Eiger detector (Dectris, Baden-Dättwil, Switzerland)
Crystal to detector distance (mm)	360
Temperature (K)	100
Wavelength (Å)	0.9537
Total/processed frames	3600
Oscillation (°)	0.1
Exposure time per frame (s)	0.2
Space group	*C*121
Cell dimensions a, b, c (Å)	213.469, 94.348, 139.831
ɑ, β, γ (°)	90, 108.163, 90.00
Resolution (Å)	50.00–3.06 (6.35–2.95)
Rpim (%)	7.4 (40.4)
Rmeas (%)	18.0 (92.7)
CC_1/2_ (%)	99.0 (74.6)
I/σ(I)	9.3 (1.3)
Completeness (%)	99.1 (96.2)
Redundancy	5.8 (4.7)
**Refinement**	
Resolution (Å)	48.20–2.94 (3.04–2.95)
Completeness (%)	97.42%
Number of reflections	55249 (1864)
Rwork/Rfree	16.28/22.28
Number of non-H atoms	14482
Protein	14167
Ligand	159
Solvent	156
Average B-factor	65.24
Macromolecules	65.25
Solvent	53.20
R.m.s. deviations	
Bond length (Å)	0.007
Bond angle (°)	0.92
Ramachandran plot	
Most favoured (%)	93.95
Allowed (%)	5.83
Outliers (%)	0.22

Statistics for the highest-resolution shell are shown in parentheses.

$R_{\mathrm{merge}}=\sum \left|I\;-\;\left\langle I\right\rangle \right|/\sum \left\langle I\right\rangle$ where I is the intensity of individual reflections.

$R_{\mathrm{fac}}=\sum_{h}\left|F_{o}-\;F_{c}\right|/\sum_{h}\left|F_{o}\right|,$ where $F_{o}$ and $F_{c}$ are the observed and calculated structure-factor amplitudes for each reflection “h”.

$R_{\mathrm{free}}$ was calculated with 5% of the diffraction data selected randomly and excluded from refinement.

### Protease activity assay

Protease activity was assessed using the EnzChek Protease Assay Kit (Invitrogen, E6638) as previously described.^36^ Briefly, all reactions were carried out using 500 nM protein in a buffer containing 50 mM Tris, 150 mM NaCl, and 0.005% Triton X-100 at pH 7.4. Fluorescence measurements were recorded at excitation/emission wavelengths of 485/520 nm using a FLUOstar Omega plate reader (BMG Labtech).

### Circular dichroism spectroscopy

Circular dichroism (CD) spectroscopy was carried out using an Aviv 420 CD spectrophotometer (USA). Wavelength scans were performed between 195 and 250 nm in a quartz cuvette with a 1.0 mm pathlength and a protein concentration of 0.2 mg mL^−1^ in PBS. The data were collected at 20°C with 0.5 nm increments and 5 s averaging time. The protein signal was normalized against the buffer signal and converted into molar ellipticity using the equation below.

Conversion of CD millidegrees into mean residue ellipticity (MRE $\left[\theta \right])$:

$$\left[\theta \right]=\mathrm{CD}\left(\mathrm{millidegrees}\right)*\frac{1}{\mathrm{MRC}*\mathrm{pathlenght}\left(\mathrm{mm}\right)}$$

Calculation of mean residue concentration (MRC):

$$\mathrm{MRC}=\frac{\mathrm{amino}\;\;a\mathrm{cid}\;\;c\mathrm{ount}}{\mathrm{MW}\left(\frac{g}{\mathrm{mol}}\right)}*\mathrm{concentration}\left(\frac{\mathrm{mg}}{\mathrm{ml}}\right)$$

Where [θ] is the mean residue ellipticity value (°cm^2^ dmol^−1^).^37^

### Thermal melts

Thermal unfolding of α^EspC^ was determined as previously described.^36^ Briefly, measurements were carried out using an Aviv 420 circular dichroism (CD) spectrophotometer (USA) which monitored the CD signal at 222 nm while increasing the temperature from 20°C to 90°C at a rate of 0.5°C/min. Experiments were conducted using 1 mm pathlength quartz cuvettes (Hellma) containing 200 μL of protein at a concentration of 0-0.2 mg mL^−1^ in PBS. The apparent melting temperature (T_m_^app^) was determined by fitting the data to the following equation:

$$y=\frac{k}{1+k}\left[\left(u+u_{1}x\right)-\left(l+l_{1}x\right)\right]+l+l_{1}x$$

Where, *y* represents absorbance, $k=e\left[\frac{h}{1.987\left(x+273.15\right)}\right]\left[\frac{x+273.15}{t+273.15}-1\right]$, *x* is temperature, *h* denotes enthalpy, *t* is T_m_^app^, *u* and *l* correspond to the folded and unfolded absorbance values, respectively, and *u*_*1*_ and *l*_*1*_ account for the linear temperature dependence of folded and unfolded states

### Cell culture

HEp-2 cells were maintained in Dulbecco’s Eagle’s medium (DMEM) (Gibco) supplemented with 10% fetal bovine serum (FBS) (Corning) and 2 mM L-glutamine (Gibco) to support optimal growth. Cultures were incubated at 37°C in a humidified atmosphere containing 5% CO₂ to mimic physiological conditions. Upon reaching approximately 80–90% confluence, cells were dissociated using trypsin-EDTA (Gibco) to detach them from the culture surface. Detached cells were centrifuged, resuspended in fresh complete DMEM, and seeded into new culture vessels at appropriate dilutions for propagation.

### Fluorescence microscopy

Activation of the T3SS^23^ in REPEC (rabbit enteropathogenic *E. coli*) was done by diluting overnight REPEC cultures (1:50) into CD-CHO media (Gibco) with no supplements, followed by overnight incubation at 37°C in 5% CO_2_. To prepare HEp-2 cells for infection, cells were seeded onto sterile 18 mm glass coverslips (Marienfeld 0,117,580) using 12-well tissue culture plates (Greiner 665,180). Each well received 1.3 × 10^5^ cells suspended in DMEM supplemented with 10% fetal bovine serum (FBS) and 2 mM L-glutamine (L-Gln). The cells were incubated for 48 h at 37°C in a 5% CO₂ atmosphere, allowing for adequate adherence and growth. Post-incubation, the cells were washed with PBS, and the medium was replaced with CD-CHO (Gibco) to align with bacterial culture conditions.

HEp-2 cells were then exposed to 100 µL of REPEC cultures adjusted to an optical density (OD₆₀₀) of approximately 0.6. Simultaneously, purified α^EspC^ proteins – either wildtype or mutant variants – were added to each well at a concentration of 60 µg. Following a 6 h infection period, cells were treated with gentamicin at a final concentration of 100 µg/mL for 1 h to eliminate extracellular bacteria. The cells were then washed thoroughly with PBS to remove any residual gentamicin and bacteria.

For microscopic analysis, cells were fixed with 4% formalin in PBS for 10 min in the absence of light, followed by washing in PBS containing 100 mM glycine. After permeabilisation with 0.2% Triton X-100 in PBS for 5 min, cells were blocked using 2% BSA dissolved in PBS containing 0.05% Tween-20 (PBS-T) for 1 h at room temperature or overnight at 4°C. The fixed and blocked cells were incubated for 2 h with rabbit polyclonal antisera specific to α^EspC^ (1:100 dilution), generated against the purified passenger domain at the Walter and Eliza Hall Antibody Facility (Australia). Secondary labeling involved Alexa Fluor Plus 647-conjugated goat anti-rabbit antibody (1:200, Invitrogen, A32733), applied for 1 h. To visualize actin structures, cells were stained with Phalloidin-iFluor 555 reagent (1:1000, Abcam, ab176756) for 30 min. Nuclei were counterstained with DAPI (1 µg/mL, Sigma, D9542) for 5 min in the dark.

Coverslips were mounted onto microscope slides using VECTASHIELD Antifade Mounting Medium (Vector Laboratories) to preserve fluorescence signals during imaging. Confocal microscopy was performed on a Zeiss LSM 780 microscope at magnifications of 10×, 40×, or 63×, depending on the required resolution. Images were processed in the FIJI^38^ using the BIOP Channel Tools plugin.

### Analytical ultracentrifugation

Sedimentation velocity analytical ultracentrifugation (SV-AUC) experiments were performed using a Beckman Optima XL-A analytical ultracentrifuge with an 8-hole An-50 Ti rotor. Double-sector quartz cells were loaded with 400 μL buffer (25 mM HEPES, pH 7.0 and 150 mM NaCl) and 480 μL α^EspC^ at 0.5, 1 and 2 mg mL^−1^. The absorbance readings were collected at 280 nm and 40,000 rpm at 20°C in continuous mode. Solvent density, solvent viscosity and estimates of the partial specific volume of α^EspC^ at 20°C were calculated with SEDNTERP. Data were fitted with a continuous-size distribution model with SEDFIT.^39^

### Galleria mellonella toxicity assay

Toxicity assays were performed in *Galleria mellonella* as previously described.^36^ Briefly, groups of seven larvae weighing approximately 180 mg each, were randomly selected and injected with 20 μL of purified α^EspC^ or α^EspC:PMSF^ with or without OD_600_ ~0.05 EPEC diluted in sterile PBS. Injections were performed using a 1 mL U-100 insulin syringe (Terumo, 29 G × 13 mm) attached to a NE-1000 syringe pump (New Era). Following the injection, the larvae were transferred to a clean plate and incubated at 37°C. Mortality was assessed daily by gently prodding the larvae with tweezers. Larvae that exhibited no movement or response to physical stimuli were recorded as dead.

## Results

### Expression and purification of EspC passenger domain (α^EspC^)

**Figure 1.**
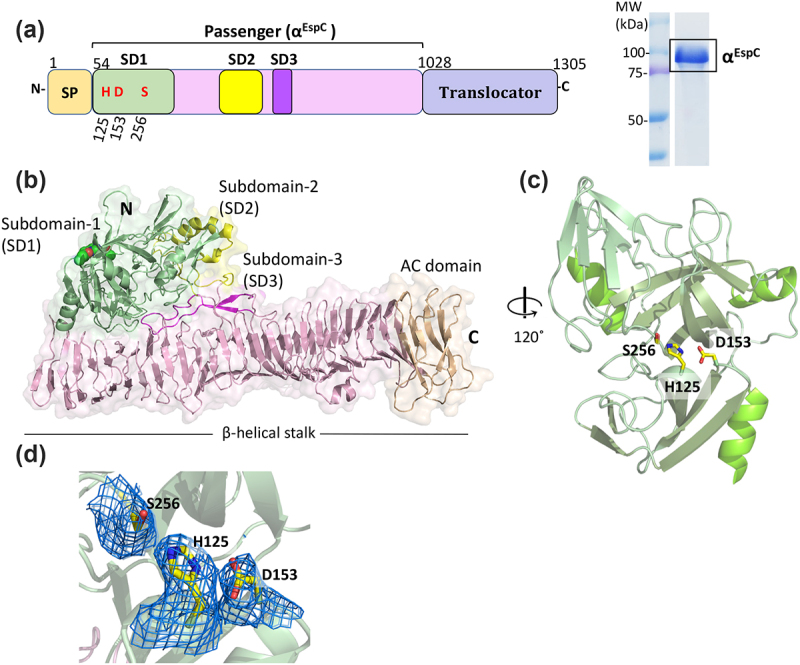
Crystal structure of EspC passenger domain (α^EspC^). (a) (left panel) linear schematic representation of EspC primary sequence, showing an N-terminal signal peptide (SP, residues 1–53), C-terminal translocator domain (residues 1029–1305) and central passenger domain (α^EspC^) (residues 54–1028), which is the functional domain and mature toxin that is released into the extracellular environment. The conserved serine protease catalytic triad is highlighted in red and includes residues Ser256, His125 and Asp153. (right panel) SDS-PAGE analysis of purified α^EspC^ showing a band at ~100 kDa, consistent with the predicted molecular weight of 104 kDa. (b) Crystal structure of the EspC passenger domain (α^EspC^) shown in cartoon representation, with the serine protease domain coloured in pale green (subdomain-1, SD1, residues 54–304) and the active side residues depicted as spheres. The right-handed β-helix stalk domain (residues 305–1028) is pink, with the C-terminal AC domain coloured in peach. The protruding ɑ-helical subdomain-2 (SD2, residues 567–620) is coloured in yellow and the β-hairpin subdomain-3 (SD3, residues 666–696) in magenta. (c) Cartoon representation of the EspC SD1 protease domain that comprises a chymotrypsin-like fold containing the catalytic triad Ser256, His256 and Asp153 (yellow sticks). (d) 2Fo-fc electron density map coloured at 1σ encompassing the close-up view of the catalytic triad residues.

Native full length *espC* from EPEC (0127:H6 E2348/69), cloned into pBAD30, was expressed in Top10 cells, which produced a 104 kDa secreted protein corresponding to the EspC functional passenger domain (α^EspC^; residues 54–1028). The culture supernatant, containing the untagged α^EspC^, was collected and concentrated using tangential flow filtration followed by purification using cation exchange and size exclusion chromatography (Figure 1(a)). The purified α^EspC^ was shown to be proteolytically active using a fluorogenic casein-based substrate (Figure S1). Further, the protease activity of α^EspC^ strongly depended on the pH and salt concentration of the medium, where the highest activity was observed at pH 9.0 and 50 mM NaCl (Figure S1).

### The overall architecture of the EspC passenger domain (α^EspC^)

The crystal structure of the passenger domain of EspC (α^EspC^) was determined by molecular replacement to a resolution of 2.9 Å, using the passenger domain of EspP (PDB: 3SZE)^30^ as a model and refined to a R_free_ of 22.28% (R _work_ 16.28%) (crystallographic statistics in Table 1). Crystals of α^EspC^ belonged to the *C*121 space group with two molecules in the asymmetric unit. However, as shown by sedimentation velocity analytical ultracentrifugation (AUC), α^EspC^ only identified as monomers in solution at concentrations up to 2 mg mL^−1^ (Figure S2). Structural alignment of the two monomers in the asymmetric unit gave an overall r.m.s.d. value of 0.429 Å over all 949 Cα atoms, indicating their high structural similarity.

Each monomer of α^EspC^ contains 949 residues (numbered 54–1002) and is arranged into two major domains, an N-terminal serine protease domain/subdomain-1 (SD1 residues 54–304; 251 aa) and a C-terminal β-helical stalk domain (β-helix residues 305–1002; 698 aa) (Figure 1(a, b)).

SD1 adopts a chymotrypsin-like fold comprised a tandem 6-stranded β-barrel architecture. The intervening surfaces of the two β-barrels harbor the protease active site residues His125, Asp153 and Ser256, with the barrels connected via an extended loop harboring a short α-helix (Figure 1(c,d)). Additional features include an extended N-terminal loop with an α-helix and β-hairpin along with a third loop derived from the second β-barrel that forms a β-hairpin to pin the N-terminus.

The large right-handed β-helix subdomain includes the auto-chaperone (AC) domain at the C-terminus spanning from Thr919 to Thr1002 (84 aa), capped by a β-hairpin motif (Figure 1(b)). The β-helix stalk comprises 24 complete turns, with each turn containing about 20 residues forming 3 β-strands linked by loops. Like other ATs, the β-helix core is largely nonpolar, while polar residues decorate the exterior solvent-accessible surface. Extensions from the core β-helical domain form two additional domains, an α-helical subdomain-2 (SD2, residues 567–620) and a β-hairpin subdomain-3 (SD3, residues 666–694) (Figure 1(a, b)). SD2 consists of a 23-residue helix-turn-helix motif adjacent to SD1 domain, and a 20-residue loop comprising two Cys residues (Cys578 and Cys585) that form a disulfide bond. Mapping parallel to the β-helix stalk is SD3, forming a long β-hairpin loop consisting of 29 residues (Figure 1(b)).

Structural comparison using the Dali server^40^ showed that α^EspC^ is most similar to class 1 SPATE passenger domains,^5^ including EspP from enterohaemorrhagic *E. coli* (41% sequence identity, PDB: 3SZE,^30^ Z-score 42, r.m.s.d. 1.8 Å over 845 Cα atoms) and Pet from enteroaggregative *E. coli* (42% sequence identity, PDB: 4OM9,^41^ Z-score 39.4, r.m.s.d. 2.2 Å over 836 Cα atoms) (Figure S3). Like α^EspC^, class 1 SPATEs contain protease and β-helix domains along with SD2 subdomain predominantly α-helical and positioned adjacent to the SD1 protease domain. In contrast, the equivalent subdomain in class 2 SPATEs, such as Hbp, adopts a chitinase-like structure, mapping in the opposite side of the β-helix relative to the protease domain, creating a distinctive Y-shaped structure.^42,43^ Additionally, class 1 SPATEs and α^EspC^ share a conserved GKNITGXGFXFRQ motif in the SD3 β-hairpin loop (Figure 3(b)), though its function is unknown.

### EspC substrate binding pocket

**Figure 2.**
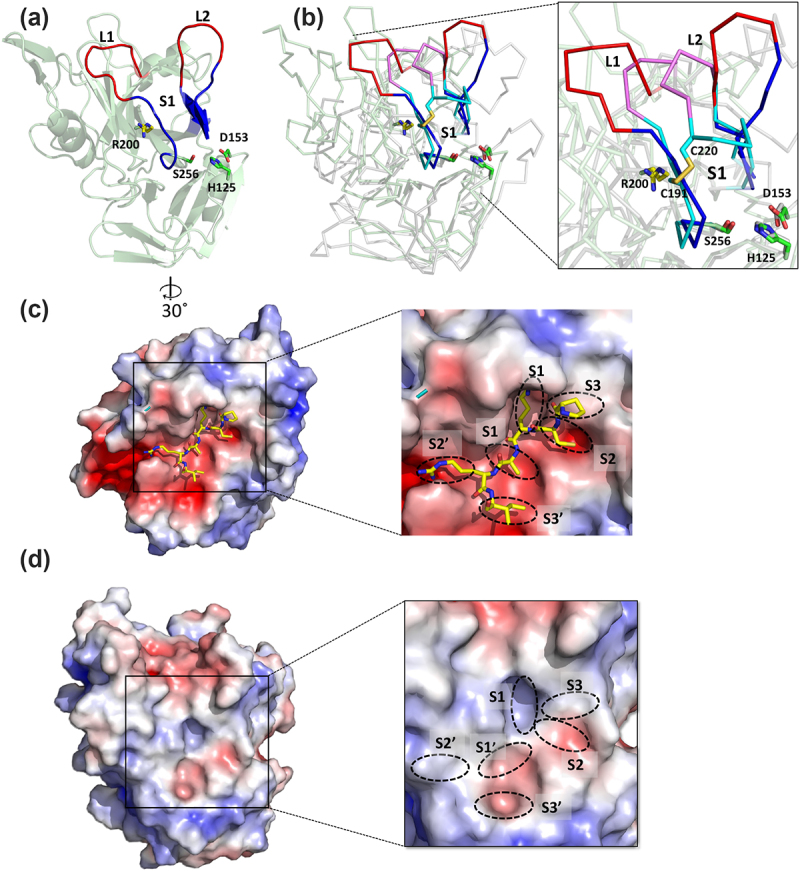
Comparison of the α^EspC^ protease domain and bovine chymotrypsin. (a) The protease domain of EspC in cartoon representation shows the catalytic triad (Ser256, His256 and Asp153) as green sticks and the S1 binding pockets in blue. The binding pocket extension loops of L1 and L2 are coloured in red, and the S1 subsite Arg200 is highlighted as yellow sticks. (b) Superimposition of the crystal structure of the protease domain of α^EspC^ (pale green) and bovine chymotrypsin in grey (PDB:1CBW). Close-up view of superimposed molecules showing the alignment of the catalytic triad of δ-chymotrypsin (Ser195, His57 and Asp102) and α^EspC^ (Ser256, His125 and Asp153). The substrate binding pocket S1 of α^EspC^ and bovine chymotrypsin are shown in blue and cyan colours, respectively. The S1 subsite of α^EspC^ (arg 200) is shown as a yellow stick. L1 and L2 loops of α^EspC^ and bovine chymotrypsin are shown in red and pink, respectively. Two cys residues of bovine chymotrypsin (C191 and C220) are shown in cyan sticks with sulphur atoms in orange. Catalytic triad residues of α^EspC^ in green (labelled) and bovine chymotrypsin in grey are shown as sticks. (c) Electrostatic surface of bovine chymotrypsin in complex with the basic pancreatic trypsin inhibitor (BPTI). The principal binding site residues (Pro13 to Ile18) of BPTI are shown as yellow sticks. Close up of subsites are labelled in insets. (d) The electrostatic surface of α^EspC^ protease domain. Putative protease subsites are indicated in the inset. The electrostatic surface potentials were calculated with the APBS plugin in Pymol with electrostatic potential coloured from negative (red) to positive (blue) with a range of ± 5 kT/e.

Serine proteases feature a substrate binding site with multiple subsites (S1–S3 and S1–S3’), with the S1 pocket serving as the primary determinant of substrate specificity.^44^ To identify the putative binding pocket of α^EspC^, and infer substrate specificities structural comparisons were made with structurally investigated serine proteases using DALI.^40^ This analysis revealed that the closest ligand-bound serine protease to α^EspC^ is bovine pancreatic δ-chymotrypsin complexed with pancreatic trypsin inhibitor (BPTI) (PDB ID: 1CBW,^45^ 16.7% sequence identity over 250 Cα atoms with r.m.s.d. of 2.0 Å). Structural superposition of the protease domains of α^EspC^ and δ-chymotrypsin-BPTI complex showed a perfect alignment of their catalytic triad residues (Figure 2(b)). This superposition also uncovered that the predicted α^EspC^ substrate binding pocket (residues 251–255, 277–281 and 287–291) is more open than that of δ-chymotrypsin, especially around the S1 subsite (Figure 2(d)). This feature is due to longer loops L1 (242–249) and L2 (282–287) near the α^EspC^ S1 binding pocket, along with the lack of a disulfide bond in this area, with a disulfide bond in chymotrypsin providing some restriction to this area (Figure 2(b)). EspC closest structural homologues Pet and EspP also have long L1 and L2 loops that lead to a large substrate binding pocket (Figure S3).

A comparison with the δ-chymotrypsin-BPTI complex revealed the complete α^EspC^ S3 to S3’ subsite positions to be arranged as h-a-b//a-h-a (S3-S2-S1//S1’-S2’-S3’; a = acidic, b = basic, h = hydrophobic). These subsites differ from the mostly acidic subsites in chymotrypsin, which are h-a-a//a-a-a (Figure 2(c)), particularly the primary S1 subsite, which in EspC harbors a positively charged Arg 200 residue.

Further comparison of the substrate binding sites of EspC with those of Pet, also determined based on its structural superposition with the δ-chymotrypsin-BPTI complex, showed notable differences between these two class 1 SPATEs. The α^Pet^ S3 to S3’ subsites were predicted to be h-a-h/a//b-b-b for S3-S2-S1//S1’-S2’-S3’, respectively, highlighting several difference including the main S1 subsite which is mostly hydrophobic in Pet and basic EspC (Figure S4).

### Extracellular secretion of α^EspC^ is independent of SD1, SD2 and SD3

**Figure 3.**
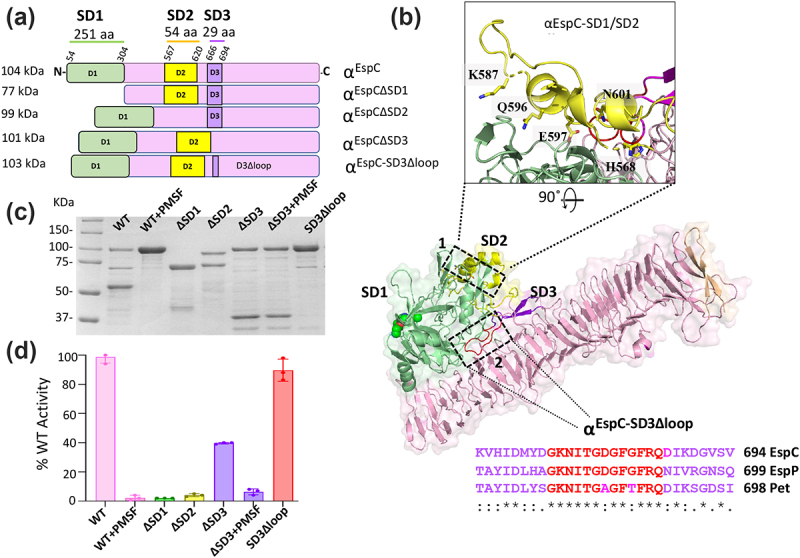
α^EspC^ variants and their protease activity (a) top diagram shows the linear schematic representation of wild type α^EspC^, highlighting SD1 (residues 55–310), SD2 (residues 575–627) and SD3 (residues 671–696). Additionally, we show α^EspC^ variants with deletions to these domains; α^EspC∆SD1^, α^EspC∆SD2^, α^EspC∆SD3^ along with α^EspC-SD3∆loop^ lacking a SD3 loop (GKNITGDGFGFRQ). (b) Cartoon representation of α^EspC^ showing SD1 (green), SD2 (yellow) and SD3 (magenta). Inset 1 shows a detailed view of the SD1-SD2 interface highlighting the residues mutated in the α^EspC-SD1/SD2^ mutant (H568A, K587A, Q596A, E597A and N601A). Inset 2 displays the sequence alignment of class 1 SPATEs α^EspC^, α^EspP^ and α^Pet^ loop regions, depicting the residues modified in the α^EspC-SD3∆loop^ mutant. (c) SDS-page analysis of active wild-type α^EspC^ and its inactivated PMSF-treated variant, as well as α^EspC^ mutants α^EspC∆SD1^, α^EspC∆SD2^, α^EspC∆SD3^, α^EspC∆SD3+PMSF^ and α^EspC-SD3∆loop^. (d) Protease activity of α^EspC^ and its mutants determined by the digestion of a fluorogenic casein substrate at 37°C. The means are shown with error bars representing the standard deviation from three replicates.

Recently, it has been shown that the SD2 of α^Pet^ plays a prominent role in host cell binding as the deletion of SD2 prevents Pet binding to the cell surface receptor cytokeratin-8 (CK-8).^14^ To determine the region(s) of α^EspC^ that facilitate internalization of α^EspC^ in the presence of T3SS, we initially generated three deletion mutants by deleting SD1 (residues 54–304), SD2 (residues 567–620) and SD3 (residues 666–694), referred to as α^EspC∆SD1^, α^EspC∆SD2^ and α^EspC∆SD3^, respectively (Figure 3(a, b)). We successfully overexpressed and purified these deletion mutants from the culture supernatant (Figure 3(c)), confirming that SD1, SD2 and SD3 are not required for secretion of α^EspC^. The correct structure of these deletion mutant proteins was supported by all of them showing a close wavelength profile (198–250 nm) and melting temperature (60–68°C) to that of wildtype EspC by circular dichroism spectroscopy (Figure S5–6).

### α^EspC^ proteolytic activity depends on subdomain-2 (SD2)

To assess the role of EspC structural subdomains in its protease activity, we compared the enzymatic activity of all α^EspC^ variants against a casein-based substrate. Deletion of the protease domain (SD1), as expected, completely abolished protease activity. Remarkably, deletion of SD2 also resulted in a dramatic reduction the protease activity of α^EspC^ compared to wild type α^EspC^ (Figure 3(d)). This was surprising given that the protease activity was thought to solely depend on the SD1 domain.^14^ SDS-PAGE analysis (Figure 3(c)) showed that the ΔSD2 variant consists of two major protein species (~100 kDa and ~75 kDa), with the full-length protein representing approximately 47% of the total sample based on densitometry. If only the full-length ΔSD2 variant retained activity and assuming its enzymatic function was unaffected by SD2 deletion, we would expect at least 40% of wild-type activity. However, the observed activity was less than 5%, indicating that SD2 plays a role possibly stabilizing the protease domain for efficient catalysis.

Analysis of all available SPATE structures showed that the SD2 is situated adjacent to the SD1 and connected via a network of inter-domain hydrogen bonds and salt bridges formed (H568 – D68, K587 – S216, Q596 – T208 and Q210, E597 – A63 and K88, in α^EspC^). To explore the role of these interactions, we generated the interface mutant α^EspC-SD1/SD2^ by substituting these residues with alanine. Unfortunately, although this mutant was secreted, it was unstable, preventing further examination. Our data, however, indicate that SD1 protease activity seems to rely on SD2, likely due to its stabilizing role, which helps maintain the protease activity of the SD1 domain.

### SD1, SD2 or SD3 are not required for α^EspC^ T3SS mediated host cell internalization

**Figure 4.**
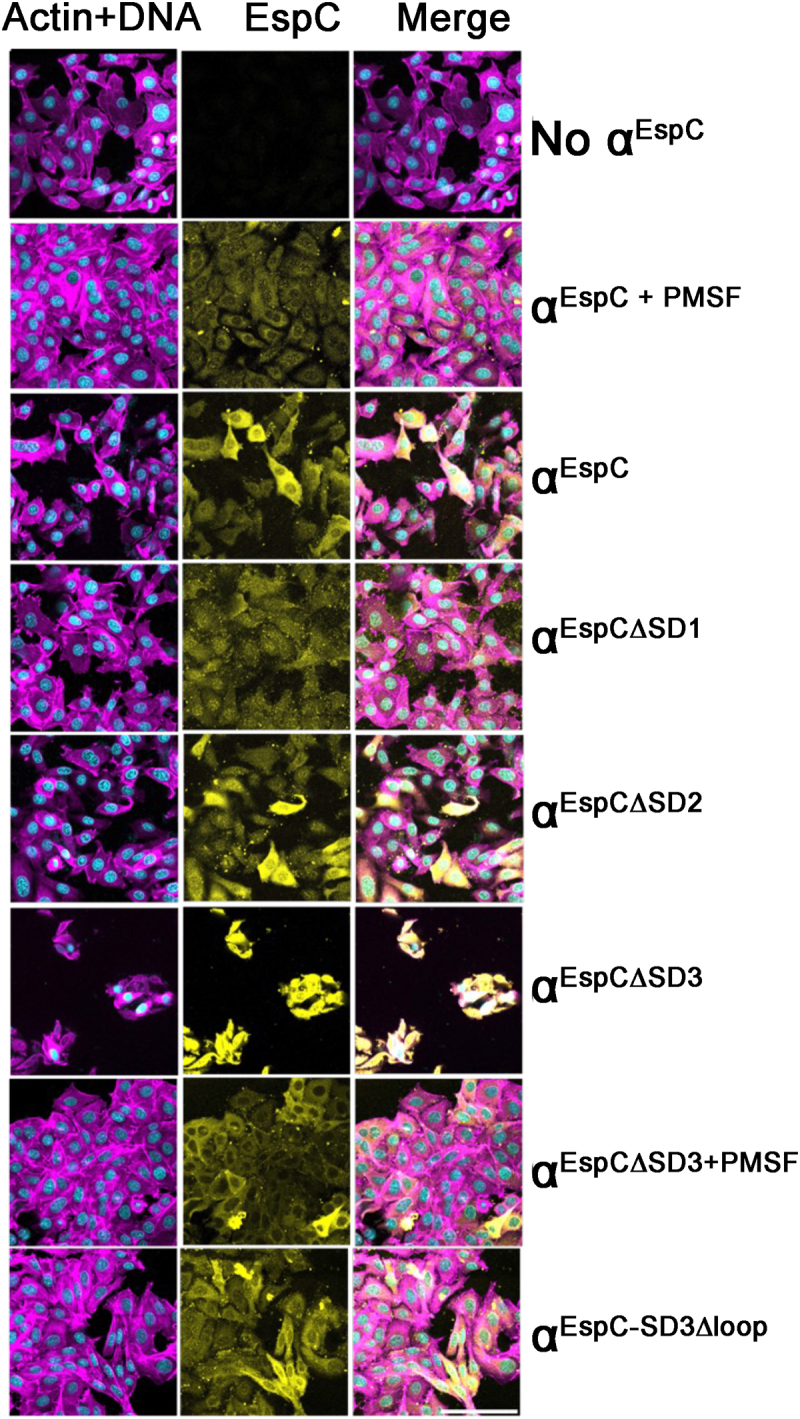
T3SS mediated α^EspC^ internalisation is independent of its major subdomains, including SD1, SD2 and SD3. HEp-2 cells were infected with REPEC and co-incubated with 60 µg of α^EspC^ variants for 6 h, followed by 1 h gentamicin treatment. The α^EspC^ was visualised with anti-α^EspC^ polyclonal antibody followed by Alexa Fluor Plus 647 conjugated secondary antibody (yellow). Actin cytoskeletal and nucleus were stained with Phalloidin (magenta) and DAPI (cyan), respectively. α^EspC^ variants include wild type (α^EspC^), deletion of SD1 (α^EspC∆SD1^), SD2 (α^EspC∆SD2^), SD3 (α^EspC∆SD3^) and a deletion of the SD3 loop motif (α^EspC-SD3∆loop^). PMSF inactivated variants were denoted by +PMSF. Images are representative of cells observed from at least three independent experiments. The scale bar represents 100 μm (lower right).

To dissect the mechanism of α^EspC^ host cell internalization, HEp-2 monolayers were incubated with purified EspC passenger domain including α^EspC^ deletion mutants (α^EspC∆SD1^, α^EspC∆SD2^ and α^EspC∆SD3^) along with native α^EspC^ and PMSF inactivated α^EspC^ (Figure 4). All EspC internalization assays were conducted in the presence of rabbit enteropathogenic *E. coli* (REPEC), which does not harbor the *espC* gene but does express a T3SS, which is required for efficient T3SS-mediated EspC internalization.^23^ Interestingly, all subdomain deletion mutants of α^EspC,^ including α^EspC∆SD1^, α^EspC∆SD2^ and α^EspC∆SD3,^ were able to enter HEp-2 cells similar to wild type α^EspC^ (Figure 4), revealing that none of these major subdomains are required for its T3SS mediated cell entry.

A detailed examination of the HEp-2 internalization assays showed that the α^EspC∆SD3^ variant exhibited higher internalization rates and caused more extensive cell damage compared to wild-type α^EspC^, as seen by increased levels of cell rounding and detachment compared to wild type α^EspC^ (Figure 4). The cell damage was attributed to the SPATE’s serine protease domain (SD1), as PMSF inactivated α^EspC∆SD3^ (Figure 4) completely abolished the cytotoxicity. However, given that α^EspC∆SD3^ did not show increased protease activity compared to the wildtype (Figure 3(d)), the increased cytoxicity was due to higher levels of α^EspC∆SD3^ penetrating into the HEp-2 cells. The conserved GKNITGXGFXFRQ motif in SD3 across class 1 SPATEs (Figure 3(b)) suggests that this subdomain plays a crucial role (Figure 3(b)). However, internalization assays with α^EspC-SD3∆loop^ mutant that contains this motif did not significantly alter internalization levels (Figure 4).

Together, these findings indicate that EspC internalization, mediated by the T3SS, does not rely on the protease activity of SD1 or the protruding SD2 and SD3, pointing to an active role of the core β-helix in facilitating cell entry.^46^ Furthermore, the removal of SD3, which maps parallel to the middle section of the β-helix (Figure S8), enhanced internalization, possibly indicating that this modification improves the β-helix’s ability to interact with the T3SS for more efficient internalization.^46^

### α^EspC^ shows higher toxicity in vivo in the presence of T3SS

Given that α^EspC^ induces cytopathic effects on human epithelial cells, we investigated its effects *in vivo* in a *Galleria mellonella* larva model, which has been used to study the cytotoxicity of the subtilase autotransporter Ssp.^36^ Purified α^EspC^ of varying concentrations was administrated by intra-hemocoel injection, either in the presence or absence of EPEC which contains the T3SS, and larvae mortality was monitored. In the absence of EPEC (T3SS), dosing of 500 mg kg^−1^ EspC (body weight) was required to cause 100% mortality, compared to PMSF-inactivated EspC, which caused no mortality at similar concentrations (Table 2). However, in the presence of 0.05 OD_600_ EPEC (T3SS), dosing of 250 mg kg^−1^ EspC showed 100% larva mortality, increasing the toxicity of EspC by two-fold (Table 2). No mortality was observed when larvae were treated with equivalent amount of EPEC (T3SS) alone. Collectively, these results highlight the importance of the α^EspC^ serine protease in causing cellular toxicity and the role of the T3SS in efficiently delivering α^EspC^ to host tissue.

**Table 2.** Percentage of surviving *G. mellonella* larvae after 24 h, post-injection with purified α^EspC^ and α^EspC:PMSF^ in the presence or absence of 0.05 OD_600_ EPEC (T3SS).

Dose (mg kg^−1^)	α^EspC^	α^EspC:PMSF^
Absence of 0.05 OD_600_ EPEC (T3SS)		
650	0%	100%
500	0%	100%
400	100%	100%
200	100%	100%
100	100%	100%
50	100%	100%
Presence of 0.05 OD_600_ EPEC (T3SS)		
400	0%	100%
250 0	0% 100%	100% 100%

## Discussion

EPEC is a serious diarrheal pathogen, particularly in infants, which produces attaching and effacing lesions in the intestinal epithelium.^17,47,48^ Here, we reveal the crystal structure of the autotransporter EspC, the first protein secreted during EPEC infection of epithelial cells,^49^ and a major enterotoxin that plays a central role in cytotoxicity, primarily via its serine protease activity.^23,50^ Upon internalization, EspC induces the degradation of key cellular components such as fodrin and focal adhesion proteins, leading to cell rounding, detachment, and eventual cell death.^23,51,52^ Although EspC uses the autotransporter system for transport to the bacterial surface, it takes advantage of the type III secretion system for injection into the intestinal epithelium.^23,53^ Guided by our crystal structure, we dissect the role of the EspC subdomains required for internalization and cell rounding of the intestinal epithelium.

Our structure of the EspC passenger domain (α^EspC^) showed strong structural conservation to other SPATEs, particularly the Class 1 SPATEs,^42^ despite the modest sequence identity (~40%). Key structural features include the long β-helix, N-terminal serine protease domain SD1, as well as the α-helical SD2 and β-hairpin SD3 domains. Given the unique ability of α^EspC^ to utilize the type III system for cell entry, it is notable that this function is linked with to the conserved core β-helix structure, conserved among autotransporters, and not to the distinctive protruding loops and domains.

The EspC protease SD1 is essential for its cytotoxicity toward epithelial cells. This aligns with previous findings on EspC and related SPATEs such as Pet, where the serine protease degrades intracellular proteins like fodrin, paxillin and focal adhesion kinase to cause cell rounding and detachment.^22,23^ However, as also shown for Pet,^14^ the serine protease activity of EspC is not required for cell entry, which contrasts with other autotransporters such as Ssp, where the protease activity is required for both cell entry and toxicity.^36^

Structurally, the α^EspC^ protease domain adopts a chymotrypsin-like fold similar to other SPATEs, but with an expanded substrate binding S1 pocket due to longer L1 and L2 loops and the absence of a tethering disulfide bond in this region. This open binding site, also conserved in EspC’s closest homologues EspP and Pet, may facilitate these proteases to cleave large oligomeric substrates, including fodrin, paxillin, and focal adhesion kinase.^25^

Mapping the putative protease binding subsites of EspC, in comparison to the δ-chymotrypsin-BPTI complex, revealed a basic electrostatic region near the S1 subsite, facilitated by Arg200. In proteases like chymotrypsin, the S1 subsite plays a pivotal role in determining substrate specificity, and EspC appears to follow a similar principle. EspC cleaves the protein fodrin at specific sites, such as LCQ/LAE and MSD/LSA,^23^ suggesting that its S1 subsite is adapted to recognize acidic or polar residues in its substrate. In contrast, while the SPATE Pet also cleaves fodrin, previous studies have demonstrated that it targets a distinct cleavage site, specifically between Met1198 and Val1199.^54^ This difference aligns with the distinct surface charge properties of Pet where the S1 subsite, is more hydrophobic and thus better suited to interact with hydrophobic residues like methionine.

Although SD2 and SD3 do not appear directly involved in EspC’s entry or cytotoxicity, they seem to play supporting roles. Removal of SD2 reduced the protease activity of EspC, suggesting that SD2 may stabilize or orient SD1 for optimal function. This partnership is possibly only required in the context of the full-length passenger domain, as isolated SPATE protease SD1, including from EspC, has been reported to retain significant protease activity.^14,46,55^ Nevertheless, the residual activity of α^EspC^ after removing the SD2 was still sufficient to generate substantial rounding of epithelial cells. SD2 function in EspC differs from the equivalent subdomain in the related Pet, which was shown to be required for cell entry.^14^ This distinction is likely due to their different cell entry mechanisms, with Pet using receptor-mediated endocytosis^56^ and EspC the type III.^24,25^

Although not critical for cell entry or toxicity, deletion of the β-hairpin SD3 appeared to increase both cell entry and cytotoxicity of α^EspC^ into epithelial cells. This enhanced cytotoxicity was attributed to the serine protease activity, as shown by the complete loss of toxicity in the PMSF-inactivated SD3 deletion mutant. However, since the SD3 deletion mutant retains only 40% of the protease activity of full-length EspC *in vitro*, the observed effects may reflect a combination of increased cell entry rates and the mutant’s inherent baseline activity. As we and others show, this cell entry depends largely on the type III secretion system.^53^ The interaction site for the type III system on α^EspC^ was previously mapped to residues 429–449,^46^ a region analogous to the translocator motif of YopH in *Yersinia*, which also depends on the T3SS for internalization.^57^ We have now identified this motif within the EspC structure, spanning six to seven β-strand rungs of the β-helix domain. The β-helix, a well-known feature of autotransporters, plays a key role in mediating protein interactions, similar to how autotransporter adhesins interact with host proteins or polysaccharides.^58,59^ Further analysis of the crystal structure reveals that the SD3 β-hairpin partially covers the type III interaction site, suggesting that SD3 regulates EspC’s interaction with the type III secretion system. Its removal seems to expose this interaction site, facilitating increased cell entry. This is the first time the role of a SPATE SD3 has been characterized. While SD3-like subdomains are common across SPATEs, they have no recorded involvement with type III systems, indicating that SD3 might serve a different, yet unidentified, role in other SPATEs.

In conclusion, our study provides important insights into the structure–function relationships of the autotransporter α^EspC^, which utilizes a type III secretion system to enter epithelial cells. We have identified distinct roles for EspC’s structural domains in mediating cell entry and cytotoxicity. Specifically, the SD1 and SD2 domains are critical for protease activity and the associated cytotoxic effects. In contrast, in its closest homologue Pet, these domains play different roles, regulating toxicity and cell entry, respectively.^14^

Although the function of the SD3 domain in autotransporters remains unclear, our findings suggest that while SD3 is not directly required for EspC internalization, it may play a regulatory role in modulating cell entry. Notably, the removal of SD3 enhances internalization, suggesting that the central region of the β-helix, partially covered by SD3, is crucial for cell entry. Its absence appears to increase both internalization and cytotoxicity, highlighting the complex interplay between structural elements and functional outcomes.

Collectively, our findings highlight the specialized features of cell-penetrating autotransporters, and provide insights into their diverse mechanisms for facilitating host cell entry and cytotoxicity.

## Supplementary Material

Supplemental Material

## Data Availability

The authors confirm that data supporting the findings of this study are available within the article or upon request from the corresponding author.

## Data deposition

The crystallography, atomic coordinates, and structure factors reported in this paper have been deposited in the Protein Data Bank under PDB ID 9MNE. The following structural models from the PDB were also used in this study: 3SZE (EspP from enterohaemorrhagic *E. coli*), 4OM9 (Pet from enteroaggregative *E. coli*), 1CBW (bovine pancreatic δ-chymotrypsin with pancreatic trypsin inhibitor).
