# Supplementary material for: The crystal structure of the toxin EspC from enteropathogenic Escherichia coli reveals the mechanism that governs host cell entry and cytotoxicity
Source: Gut Microbes. 2025 Mar 31;17(1):2483777. doi: 10.1080/19490976.2025.2483777 (PMC11970781; doi:10.1080/19490976.2025.2483777)
Supplement: Supplemental Material [file KGMI_A_2483777_SM9560.docx]

## SUPPORTING INFORMATION


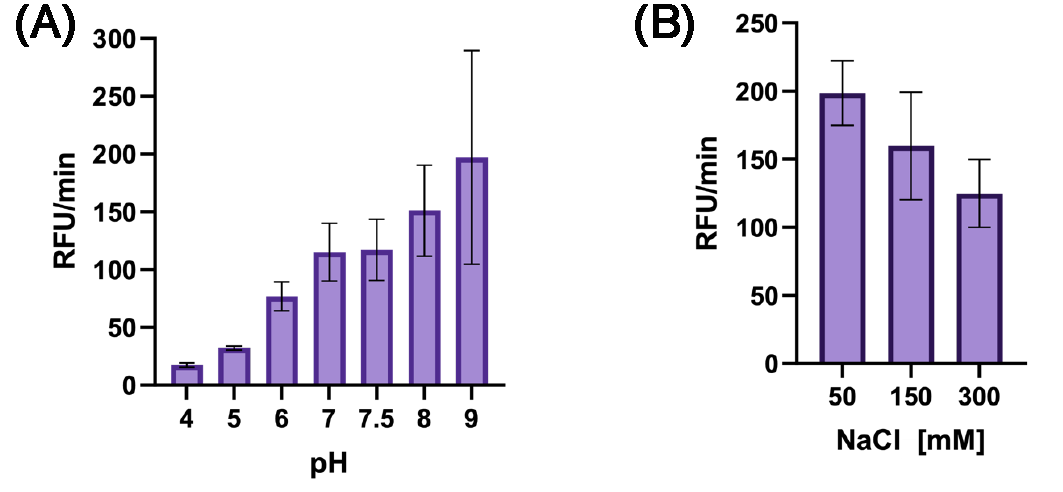


**Figure S1. Characterisation of αEspC protease activity.** Protease activity of αEspC was determined by the digestion of a fluorogenic casein substrate at 37°C (A) against seven different pH values, including pH 4.0 and 5.0 in 25 mM sodium acetate and pH 6.0 to 9.0 in 25 mM HEPES buffer at a constant salt concentration (150 mM NaCl) (B) against three different salt concentrations (50, 150 and 300 mM NaCl) in 25 mM HEPES at pH 8.0. The means are shown with error bars representing the standard deviation from three replicates.


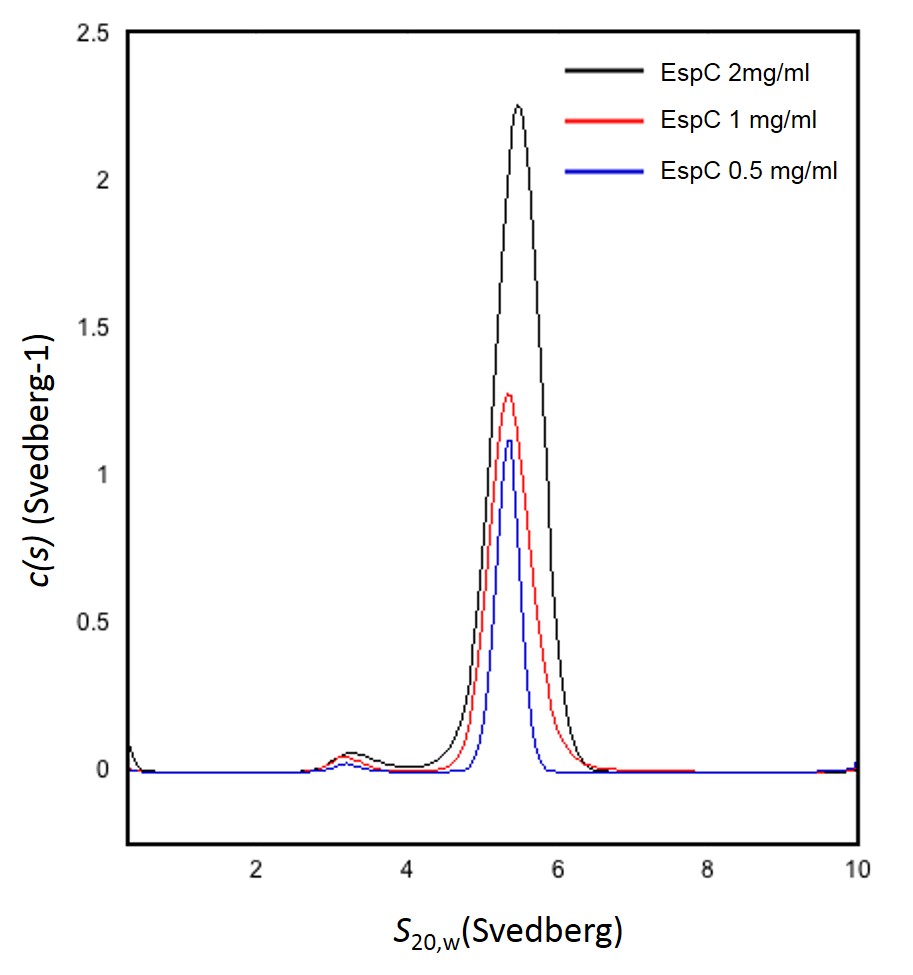


**Figure S2. Analytical ultracentrifugation of the** **αEspC**. Shown is a continuous sedimentation coefficient [c(s)] distribution analysis of a sedimentation velocity experiment performed on the αEspC at concentrations of 0.5, 1.0 and 2.0 mg mL^-1^ in 25 mM HEPES, 150 mM NaCl, pH 7.0. The distribution produced a single sedimentation boundary and a continuous sedimentation-coefficient distribution [c(s)] to give a single species with a standardised sedimentation coefficient of 5.4 S. Analysis by continuous mass distribution (*c*(*M*)) gave a molecular weight of approximately 93 kDa, consistent with a monomeric species.


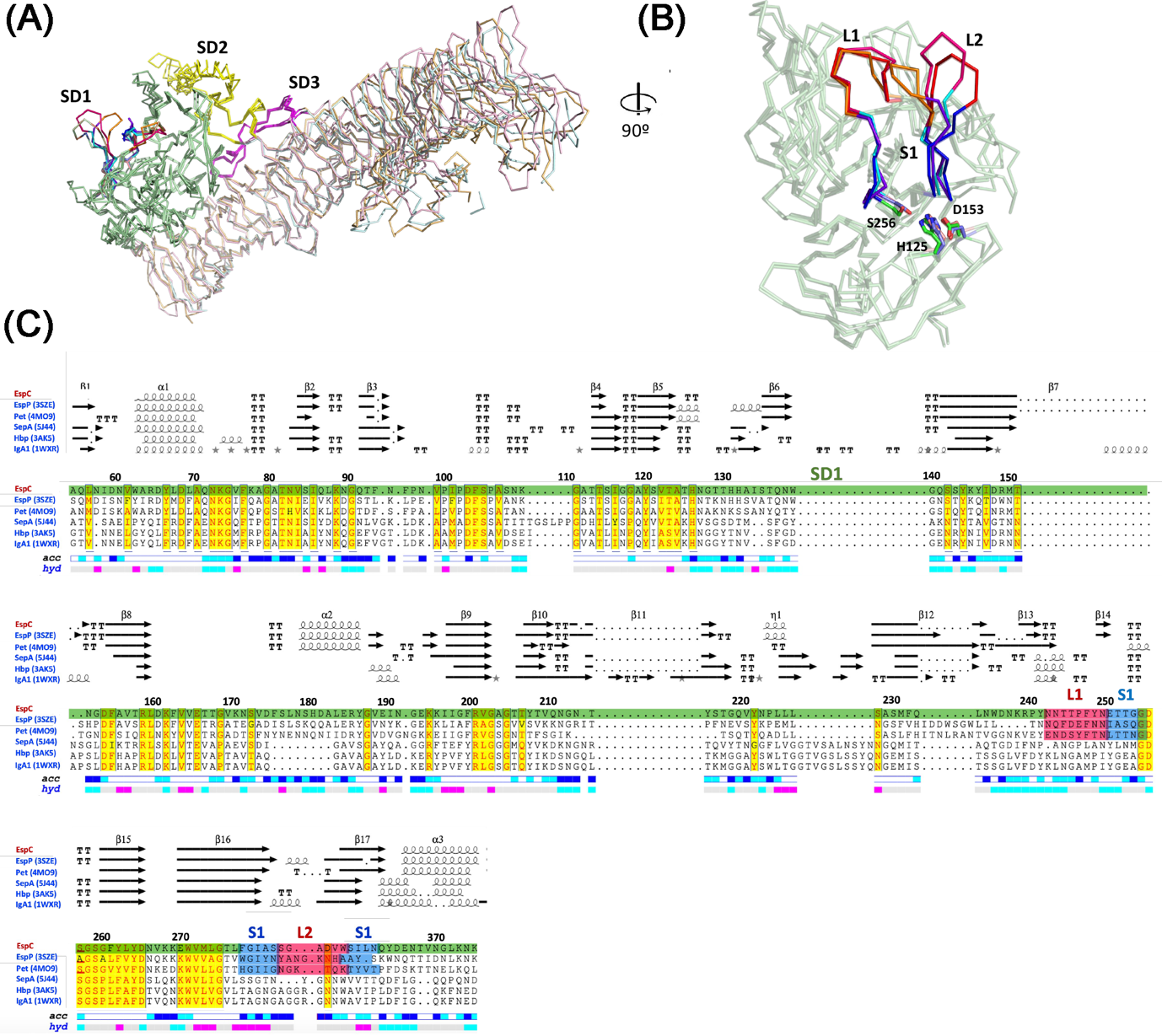


**Figure S3. Structure-based sequence alignment of αEspC and other SPATEs. (**A) Superimposition of the crystal structures of the αEspC (light pink), αEspP (PDB:3SZE)^1^ (light orange) and αPet (PDB:4OM9)^2^ (Cyan), highlighting the αEspC SD1 in green, SD3 in yellow and SD4 in magenta along with the αEspP SD1, SD3 and SD4 in dark grey and αPet SD1, SD3 and SD4 in light grey. (B) Closeup view of the protease domain overlay of αEspC, αEspP and αPet showing, substrate binding pocket (S1) in blue, purple and cyan, respectively (C) Sequence alignment of SPATEs protease domain highlighting αEspC S1 binding pocket and L1, L2 loops using the same colour code as (A) and (B).


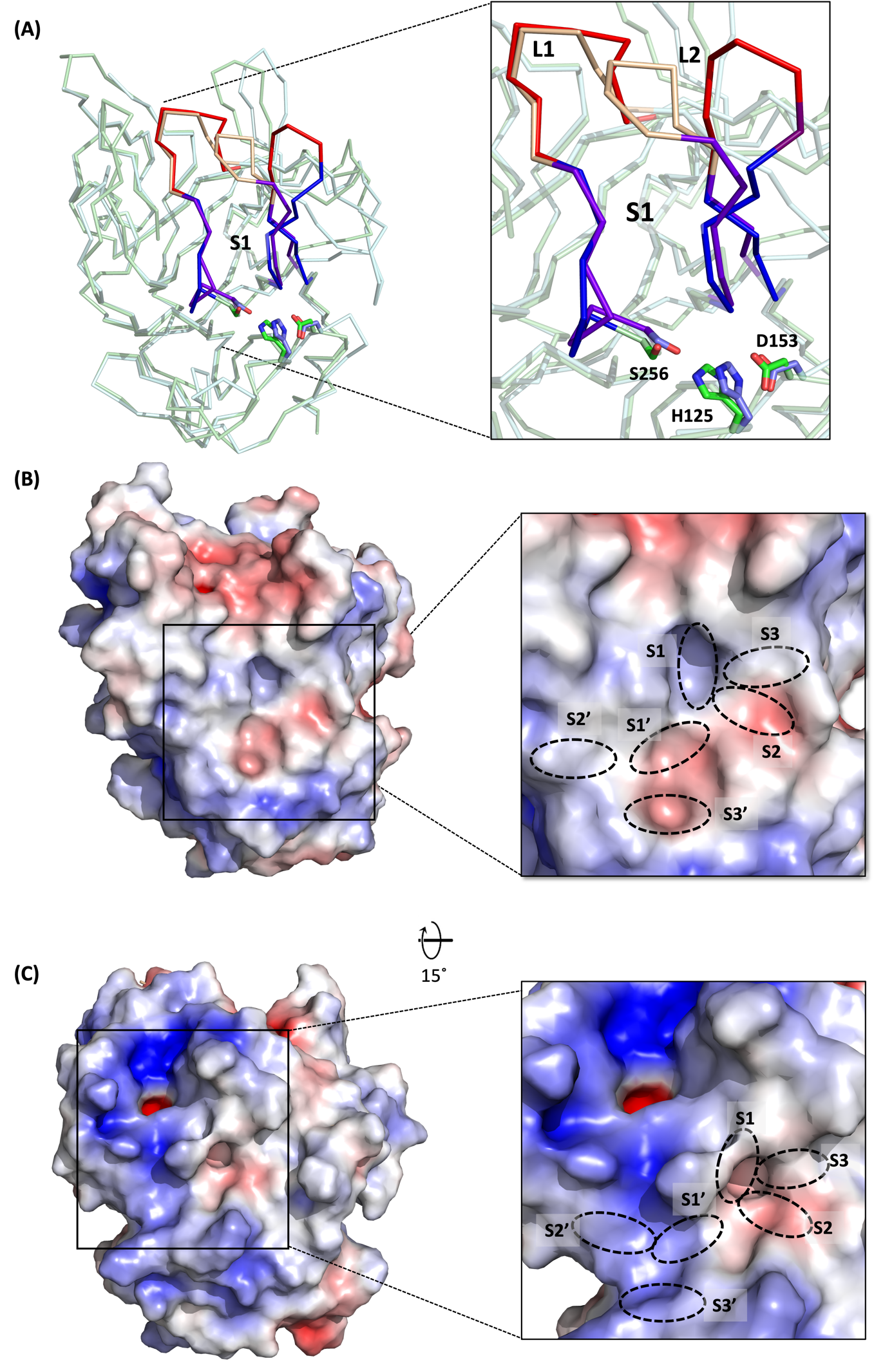


**Figure S4. Comparison of the αEspC and αPet protease domains** (A) Superimposition of the crystal structure of the protease domain of αEspC (pale green) and αPet (PDB:4OM9)^2^ in pale cyan. Close-up view of superimposed molecules showing the alignment of catalytic triad of αEspC (Ser256, His125 and Asp153) in green (labelled) and αPet (Ser260, His124 and Asp153) in light purple as sticks. The substrate binding pocket S1 of αEspC and αPet are shown in blue and purple colours, along with L1 and L2 loops of αEspC and αPet in red and tan respectively. (B) Electrostatic surface of αEspC shows its close up of subsites in inset. (C) The electrostatic surface of αPet protease domain. Putative protease subsites are indicated in the inset. The electrostatic surface potentials were calculated with the APBS plugin in Pymol with electrostatic potential coloured from negative (red) to positive (blue) with a range of ± 5 kT/e.


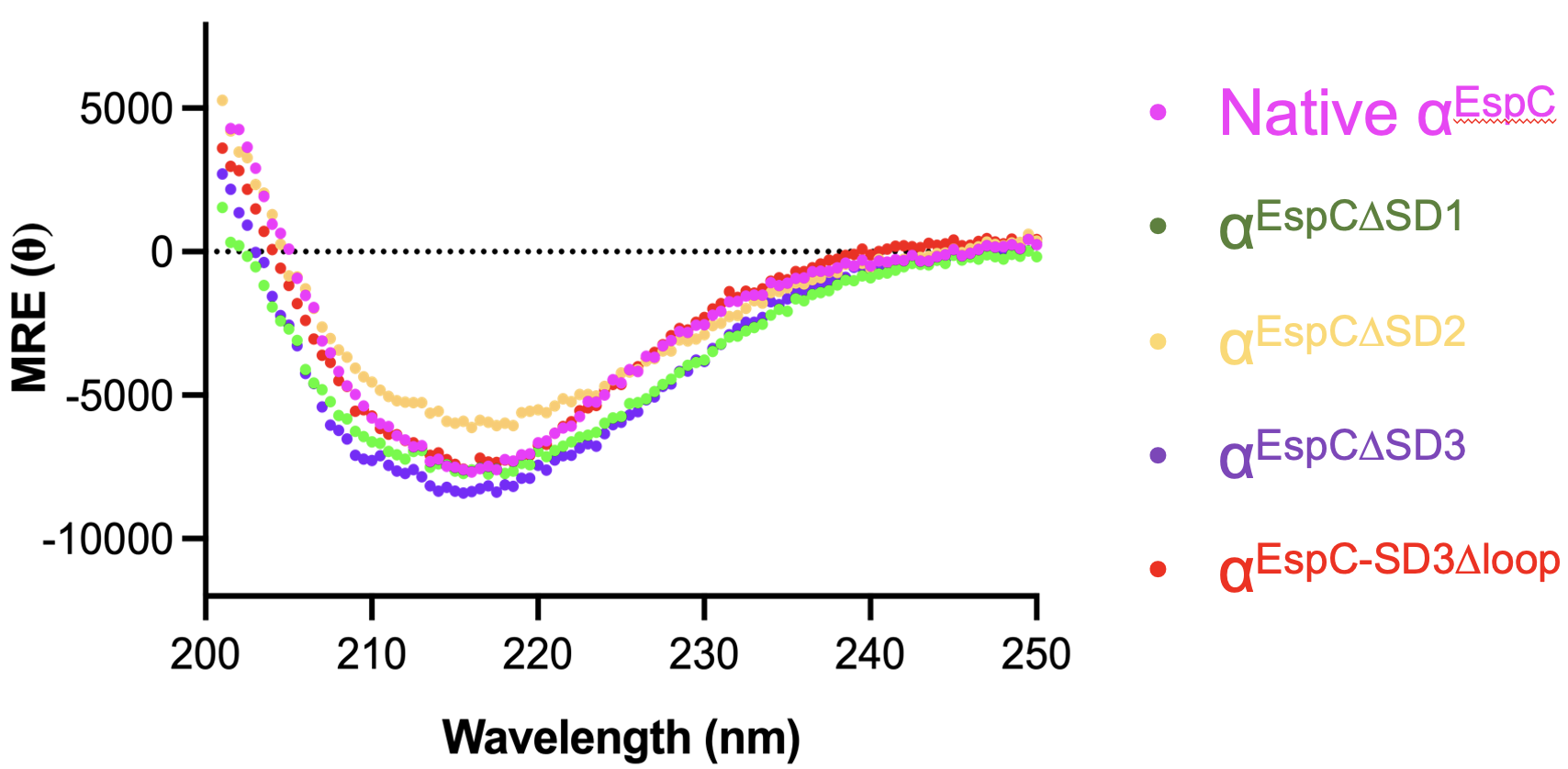


**Figure S5. Circular dichroism wavelength scans of αEspC variants.** Wavelength scans from 198–250 nm were performed on 0.2 mg mL^-1^ native αEspC, and its mutants (αEspC∆SD1, αEspC∆SD2, αEspC∆SD3 and αEspC-SD3∆loop) in PBS.


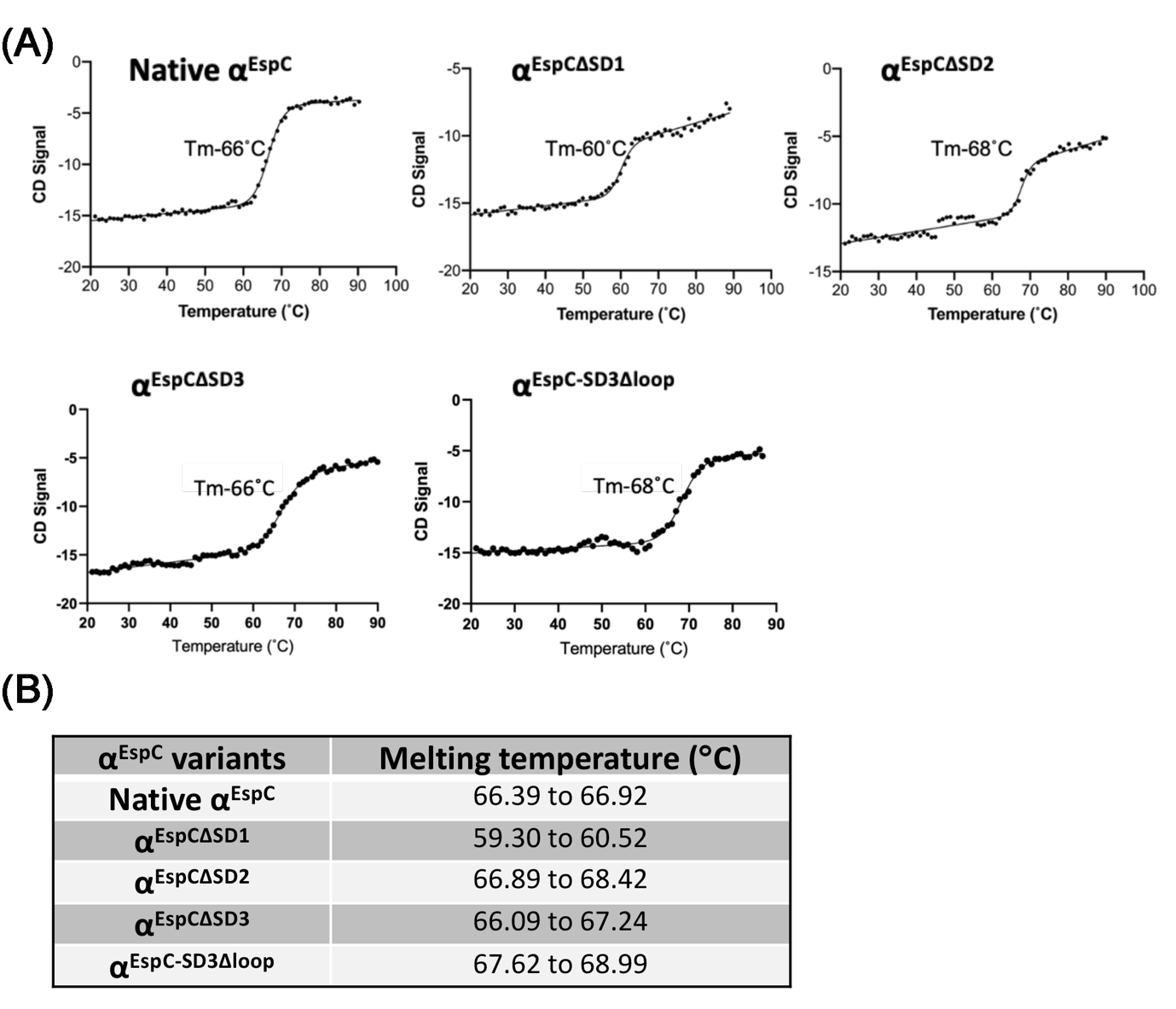


**Figure S6. Thermal unfolding of αEspC variants.** (A) Circular dichroism determined melting curves of native αEspC and αEspC variants at ~0.2 mg ml^-1^ in PBS from 20 °C to 90 °C measured at 218 nm. (B) Table summarising all determined temperatures.


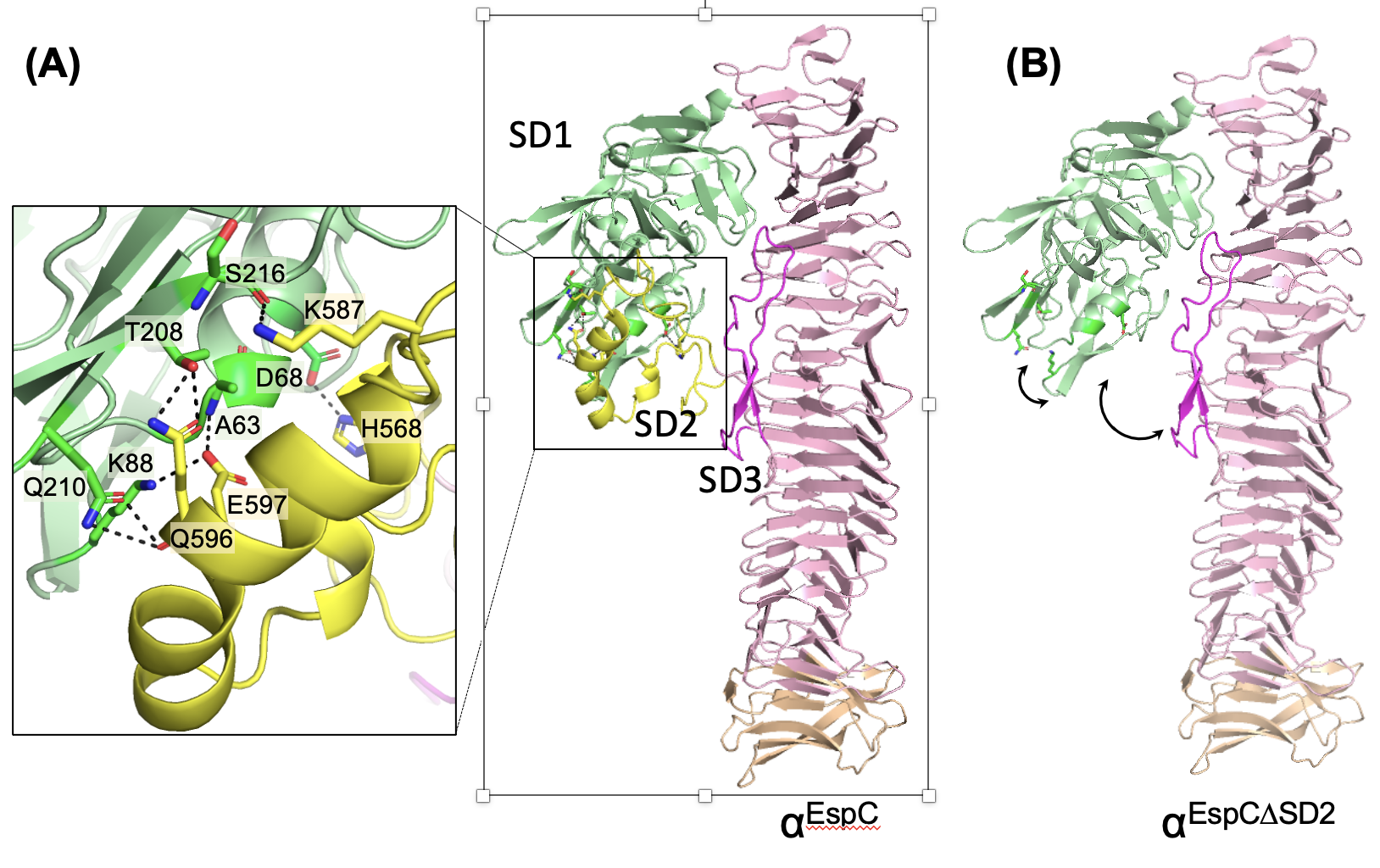


**Figure S7. (A)** Cartoon representation of αEspC showing the β-helix (pink) harbouring the SD1 (green), SD2 (yellow) and SD3 (magenta) domains. Inset shows a detailed view of the SD1-SD2 interface highlighting key interactions that brace SD1 to SD2 and, consequently, to the β-helix (H568–D68, K587–S216, Q596–T208/Q210, and E597–A63/K88). (B) A model of αEspC∆SD2 variant which lacks SD2, illustrating how the removal of SD2 disrupts the bracing, increasing the flexibility of the protease domain relative to the β-helix and likely leading to a less stable SD1 domain. This destabilization could result in a lower proportion of fully folded, active protein and reduced protease efficiency due to increased conformational flexibility. These structural changes likely contribute to the observed decrease in protease activity in the absence of SD2

**
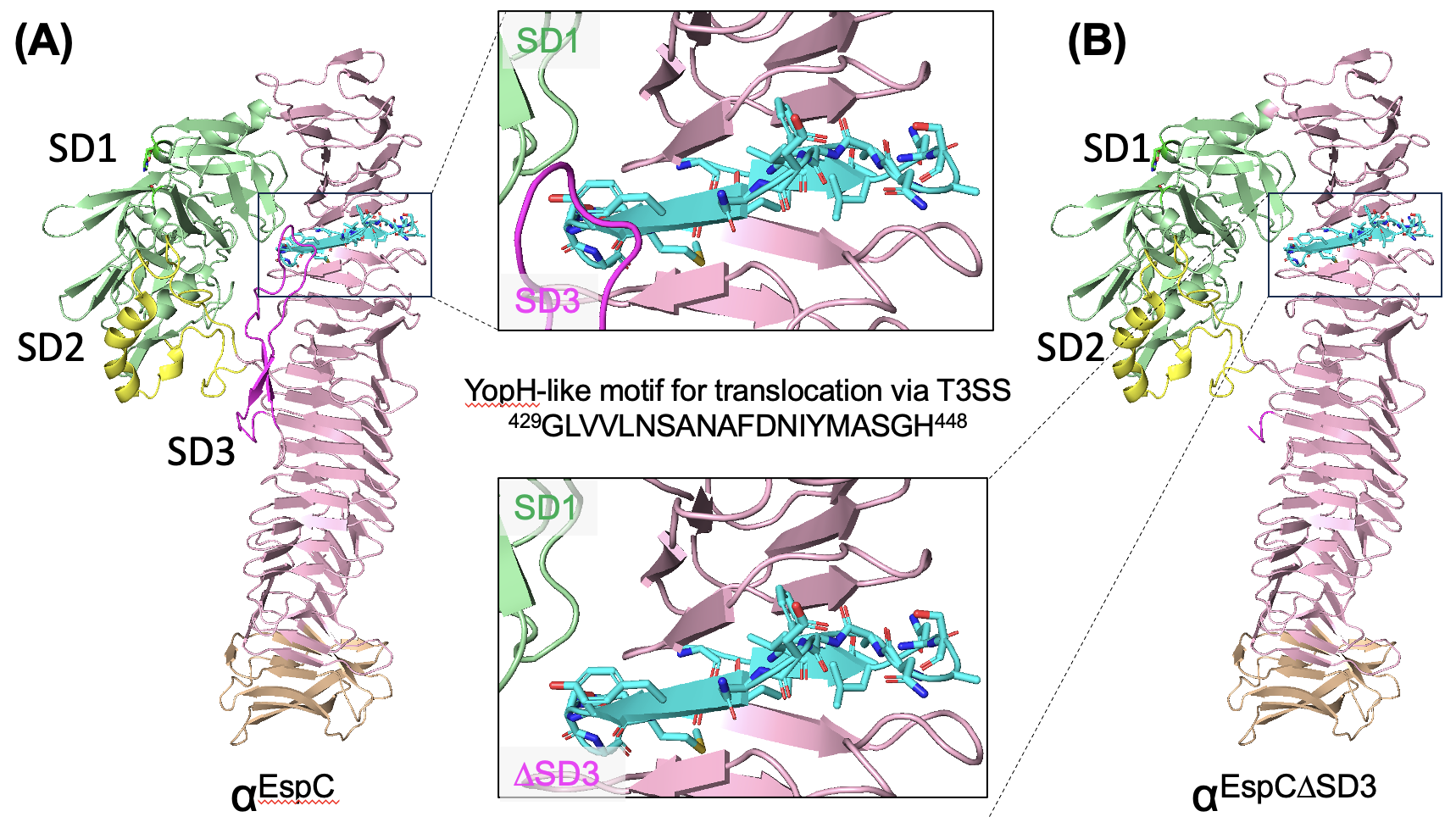
**

**Figure S8. EspC translocation motif.** (A) The crystal structure of αEspC highlighting the β-helix (pink) along with the SD1 (green), SD2 (yellow), and SD3 (magenta) domains. Indest shows the YopH-like motif (cyan), located within the β-helix, which is required for EspC translocation via the T3SS. Previous studies have shown that EspC binds to EspA filaments, travels to the EspD/EspB translocator pore, and enters host cells. (B) A model of αEspC∆SD3 variant Our structural data indicates that the SD3 domain is positioned adjacent to the β-helix stalk, partially covering the translocation motif. Deletion of SD3 enhances EspC internalisation, likely by uncovering this motif and enhancing binding to the T3SS.

**Table S1. Bacterial strains and plasmids used in this study**

| **Strains** | **Characteristics** | **References** |
| --- | --- | --- |
| REPEC 83/39 Rif ^R^ | LEE+ espC mutant (*espC* ^-^) | 3 |
| EPEC | Prototype EPEC isolate (O 127:H6), *es*pC^+^ | Pearson Lab |
| **Plasmids** | | |
| pBAD30:*espC (*pJLM174) | Native *espC* cloned into pBAD30 (Ampr) (EspC WT) | 4 |
| pBAD30:*espC∆SD1* | EspC∆SD1 derived from pBAD30:*espC*  (EspC subdomain-1 deletion) | This study |
| pBAD30:*espC∆SD3* | EspC∆SD3 derived from pBAD30:*espC*  (EspC subdomain-3 deletion) | This study |
| pBAD30:*espC∆SD4* | EspC∆SD4 derived from pBAD30:*espC*  (EspC subdomain-4 deletion) | This study |
| pBAD30:espC-SD4∆Loop | EspC-SD4*∆loop* derived from pBAD30:*espC*  (EspC-SD4 conserved loop deletion) | This study |
| pBAD30:espC-SD1/SD3 | EspC-SD1/SD3 derived from pBAD30:*espC*  (EspC-SD1/SD3 interface mutant) | This study |

**Table S2. Oligonucleotides used in this study**

| **Generated plasmids/mutants** | **Primers (5’-3’)** | **Reference** |
| --- | --- | --- |
| pBAD30:*espC∆SD1* | F: ACTCAAAAAGTCCAGTTAAACAATAATAC  R: AGCCTGAGATGCGCTTAA | This study |
| pBAD30:*espC∆SD3* | F: GACTGGGAAAATCGTCTG  R: TGTAGCATGTCCCTGCAT | This study |
| pBAD30:*espC∆SD4* | F: TCTCCTGAGAGCAGCAGT  R: TGTATCTTTTCCACTTAAAGACAG | This study |
| pBAD30:espC-SD4∆loop | F: CAGGATATTAAAGATGGTGTATC  R: GCCGTCATACATATCAATATG | This study |
| pBAD30:espC-SD1/SD3 | F1: CTACATCTGCTGCTGTTTTTAGAGAGGGTGGG  F2: CAAGCCGCAAACTCAGCCGCTAAAAATAATAATACAG  R1: CATGTCCCTGCATGGTGAGCTGG  R2: CTGTATGCCTGAAACATAATCCGCTTCACAAATAAC | This study |

**Table S3. αEspC data collection and refinement statistics**

| **Parameter** | **Native αEspC** |
| --- | --- |
| **Data collection** | |
| Detector | Eiger detector (Dectris, Baden-Dättwil, Switzerland) |
| Crystal to detector distance (mm) | 360 |
| Temperature (K) | 100 |
| Wavelength (Å) | 0.9537 |
| Total/processed frames | 3600 |
| Oscillation (°) | 0.1 |
| Exposure time per frame (s) | 0.2 |
| Space group | *C*121 |
| Cell dimensions a, b, c (Å) | 213.469, 94.348, 139.831 |
| ɑ, β, 𝛾 (°) | 90, 108.163, 90.00 |
| Resolution (Å) | 50.00–3.06 (6.35–2.95) |
| Rpim (%) | 7.4 (40.4) |
| Rmeas (%) | 18.0 (92.7) |
| CC_1/2_ (%) | 99.0 (74.6) |
| I/σ(I) | 9.3 (1.3) |
| Completeness (%) | 99.1 (96.2) |
| Redundancy | 5.8 (4.7) |
| **Refinement** | |
| Resolution (Å) | 48.20–2.94 (3.04–2.95) |
| Completeness (%) | 97.42% |
| Number of reflections | 55249 (1864) |
| Rwork/Rfree | 16.28/22.28 |
| Number of non-H atoms | 14482 |
| Protein | 14167 |
| Ligand | 159 |
| Solvent | 156 |
| Average B-factor | 65.24 |
| Macromolecules | 65.25 |
| Solvent | 53.20 |
| R.m.s. deviations |  |
| Bond length (Å) | 0.007 |
| Bond angle (°) | 0.92 |
| Ramachandran plot |  |
| Most favoured (%) | 93.95 |
| Allowed (%) | 5.83 |
| Outliers (%) | 0.22 |
| Statistics for the highest-resolution shell are shown in parentheses.  R_merge_ = ∑\|I - <I>\|/∑<I> where I is the intensity of individual reflections.  R_fac_ = ∑_h_\|F_o_ - F_c_\|/∑_h_\|F_o_\|, where F_o_ and F_c_ are the observed and calculated structure-factor amplitudes for each reflection “h”.  R_free_ was calculated with 5% of the diffraction data selected randomly and excluded from refinement. | |
